# Factors affecting macromolecule orientations in thin films formed in cryo-EM

**DOI:** 10.1107/S2059798324005229

**Published:** 2024-06-27

**Authors:** Swati Yadav, Kutti R. Vinothkumar

**Affiliations:** a National Centre for Biological Sciences Tata Institute of Fundamental Research GKVK Post, Bellary Road Bengaluru560 065 India; University of Cambridge, United Kingdom

**Keywords:** cryo-EM, thin films, preferred macromolecular orientation, surfactants, temperature

## Abstract

The formation of a vitrified thin film embedded with randomly oriented macromolecules is an essential prerequisite for cryogenic sample electron microscopy. Most commonly, this is achieved using the plunge-freeze method first described nearly 40 years ago. Although this is a robust method, the behaviour of different macromolecules shows great variation upon freezing and often needs to be optimized to obtain an isotropic, high-resolution reconstruction. For a macromolecule in such a film, the probability of encountering the air–water interface in the time between blotting and freezing and adopting preferred orientations is very high. 3D reconstruction using preferentially oriented particles often leads to anisotropic and uninterpretable maps. Currently, there are no general solutions to this prevalent issue, but several approaches largely focusing on sample preparation with the use of additives and novel grid modifications have been attempted. In this study, the effect of physical and chemical factors on the orientations of macromolecules was investigated through an analysis of selected well studied macromolecules, and important parameters that determine the behaviour of proteins on cryo-EM grids were revealed. These insights highlight the nature of the interactions that cause preferred orientations and can be utilized to systematically address orientation bias for any given macromolecule and to provide a framework to design small-molecule additives to enhance sample stability and behaviour.

## Abstract 2

Preferred orientation of macromolecules is one of the major issues that are commonly encountered in obtaining isotropic cryo-EM maps. Here, a comprehensive examination was performed of how macromolecule orientations respond to changes in physical factors, such as freezing temperature, and chemical factors, such as the addition of surfactants, for a standard set of macromolecules, which provides insights into their behaviour on grids and can be utilized to address the preferred orientation problem in a systemic manner for any given macromolecule.

## 1. Introduction

Single-particle cryo-EM has become an indispensable technique in structural biology owing to its relative ease of image acquisition, reconstruction and structure determination. Consequently, there has been a steady increase in the number of structures deposited in the Electron Microscopy Database (EMDB) in the past few years (Patwardhan, 2017 ▸). Near-atomic resolution maps of biological macromolecules can now routinely be obtained due to improved hardware and specimen-preparation methods along with developments in algorithms for image processing (Bai, 2021 ▸; Cheng, 2015 ▸; Chua *et al.*, 2022 ▸; Lyumkis, 2019 ▸; Nogales, 2016 ▸; Vinothkumar & Henderson, 2016 ▸; Zheng *et al.*, 2023 ▸). Obtaining a thin film of purified macromolecules in a vitreous layer of ice transparent to electrons is an important first step in cryo-EM structure determination. Currently, the plunge-freeze method is the most commonly used technique for specimen preparation. This method involves placing a drop of the sample onto a plasma-cleaned metal grid (typically coated with carbon and having an array of holes), on which a thin film is formed as the excess fluid is blotted away using blotting paper (Dubochet *et al.*, 1982 ▸; Dubochet & McDowall, 1981 ▸). The grid is then plunged into a cryogen, causing vitrification of the water and preserving the native structures of macromolecules (Dubochet *et al.*, 1985 ▸; Dubochet & McDowall, 1981 ▸; Taylor & Glaeser, 1974 ▸).

The freezing and blotting conditions must be extensively optimized for temperature and humidity in order to reproducibly create a single layer of macromolecules on a thin film of ice suitable for imaging. Because of their large size, the first test specimens used to optimize these conditions were liposomes and viruses. They lose their structural integrity if the osmolarity of the buffer changes owing to slow cooling (Adrian *et al.*, 1984 ▸; Dubochet *et al.*, 1988 ▸). It has been shown through recent studies that during freezing, macromolecules can very often interact with the air–water interface (AWI). This interaction can cause the macromolecules to denature, dissociate, adopt preferential orientations or avoid the holes completely and stick to the carbon support (D’Imprima *et al.*, 2019 ▸; Drulyte *et al.*, 2018 ▸; Kampjut *et al.*, 2021 ▸; Liu & Wang, 2023 ▸; Noble, Dandey *et al.*, 2018 ▸; Noble, Wei *et al.*, 2018 ▸). Along with protein biochemistry, the propensity of macromolecules to adopt preferential orientations may be a common rate-limiting step in high-resolution structure determination using cryo-EM.

Depending on the instrumental setup, the time between blotting and freezing can vary, but it is typically around a second. During this period, the macromolecules are suspended in an extremely thin film and constantly tumble in solution, undergoing Brownian motion. An intact macromolecule undergoing diffusion can encounter and become trapped in specific orientations at the freshly formed AWI. This effect is protein-dependent and is more severe in some cases than in others (Naydenova & Russo, 2017 ▸).

**Figure 1:**
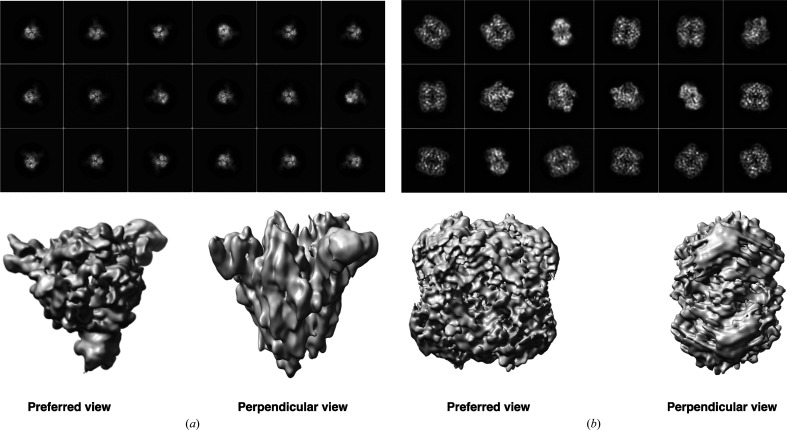
Examples of anisotropic cryo-EM maps resulting from orientation bias. The upper panel shows the reference-free 2D class averages of (*a*) SARS-CoV-2 spike protein and (*b*) human erythrocyte catalase. For the spike protein, preferred bottom views are observed. In the case of catalase, a preference for the top/bottom view is evident. In the lower panel, 3D maps with anisotropic features are shown for the preferred and perpendicular views as labelled. The symmetries applied during reconstruction were *C*1 and *D*2 for the spike protein and catalase, respectively.

When and how does having preferred orientations become a problem? There are cases in which a preferred orientation exists but does not pose a problem, such as in proteasomes and viruses because of their highly symmetric architecture (Campbell *et al.*, 2015 ▸; Vogel *et al.*, 1986 ▸). A characteristic feature of maps reconstructed from particles with orientation bias is a stretching of density in the cryo-EM map in one direction (anisotropy; Fig. 1 ▸), which makes the maps uninterpretable (Sorzano *et al.*, 2022 ▸). In such cases, the resolution estimate based on comparison of the half-maps is unrealistic and the features in the map do not justify the resolution. Two examples of macromolecules exhibiting orientation bias are shown in Fig. 1 ▸. It should be noted that 2D projections of different views of a macromolecule contribute differently to the final reconstruction, and this is an important criterion for obtaining a reconstruction with isotropic resolution, as shown by Tan *et al.* (2017 ▸) and further demonstrated using catalase as an example in Supplementary Fig. S1.

Several approaches have been proposed to address the preferred orientation problem (Drulyte *et al.*, 2018 ▸). One such approach is to change the sample-preparation process by skipping the blotting with filter papers altogether and minimizing the time between thin-film creation and freezing to as little as 100 ms. This method uses picolitre to nanolitre quantities of the sample, which is sprayed onto a self-wicking grid that contains nanowires to absorb the excess liquid, creating a thin film, followed by rapid freezing of the grid. This protocol has been shown to introduce views in some proteins; however, even in these cases the timescale of 100 ms is long enough to allow the formation of denatured protein films on the surface (Dandey *et al.*, 2020 ▸; Jain *et al.*, 2012 ▸; Wei *et al.*, 2018 ▸). Other options include using support films, such as carbon and functionalized graphene, or tilting the stage to obtain alternate views (Aiyer *et al.*, 2024 ▸; Lu *et al.*, 2022 ▸; Noble, Wei *et al.*, 2018 ▸; Tan *et al.*, 2017 ▸; Xu *et al.*, 2021 ▸). Tilting the stage or the use of a carbon support film leads to a decrease in the signal-to-noise ratio, especially if the protein is small, as the contrast is decreased due to either the support film or thick ice, while imaging at higher tilt angles and tilting is less commonly used. While carbon support films are more commonly used in samples such as ribosomes, viruses and some ion channels (Baker *et al.*, 2021 ▸; Hesketh *et al.*, 2018 ▸; Tobiasson *et al.*, 2022 ▸), graphene/graphene oxide is suitable for low-molecular-weight specimens. These techniques have been successful in alleviating orientation bias in a few cases, but are not generally applicable. Additionally, some image-processing software now deals with orientation bias in the data better than other software because of improved weighting of the different projections during reconstruction, but the problem still persists (Ramírez-Aportela *et al.*, 2022 ▸; Sorzano *et al.*, 2021 ▸).

The most widely used approach in decreasing the preferred orientation of macromolecules is to add surfactants to the sample buffer prior to grid preparation (Chen *et al.*, 2019 ▸, 2022 ▸; Li *et al.*, 2021 ▸). This method is popular because it is simple and does not require any additional steps, techniques or instruments. A large number of surfactants are available and can be used, but care must be taken to avoid disrupting the native structure of the protein in the process. Often, extensive trial and error is involved when surfactants are screened to overcome the preferential orientation problem. The EMDB contains at least 83 different soluble protein structures (retrieved in 2022) in which a detergent has been added to the buffer. The most popular choices of detergent are non-ionic or zwitterionic. Recently, cationic or anionic detergents have been found to work for a few samples, but their use has not yet been generalized (Li *et al.*, 2021 ▸). We also encountered the preferred orientation problem in many of our projects. We therefore asked whether an informed decision on the choice of grid-freezing conditions can be made based on the properties of the protein, thus minimizing the time spent screening many surfactants and specimen-preparation conditions.

To achieve this goal, we tested some commonly used surfactants with different properties on a set of five proteins: C-reactive protein (CRP) pentamers, CRP decamers, catalase, PaaZ and spike. In addition, we explored the effect of the presence of the histidine tag for spike and β-galactosidase and of physical factors such as the temperature during the sample-application step for catalase and PaaZ. We also serendipitously observed an effect of the grid hole dimensions of the holey carbon grid on the orientation distribution of catalase and discuss this briefly. Through this analysis, we identified factors that affect and determine the behaviour of the macromolecule on grids before freezing and studied their effects with a focus on the preferred orientation problem. This account highlights the factors that contribute to orientation bias and provides valuable information that can assist in achieving the optimal freezing conditions for any given macromolecule.

## 2. Materials and methods

### 2.1. Source of proteins

Human C-reactive protein (catalogue No. C4063) and human erythrocyte catalase (catalogue No. C3556) were obtained from Sigma–Aldrich. The protein samples were either concentrated using an Amicon 100 kDa concentrator or diluted in respective buffers for grid freezing. All detergent stocks were made in ultrapure water and dilutions were made and used on the day of the experiment.

PaaZ was expressed and purified as described in Sathyanarayanan *et al.* (2019 ▸).

The SARS-CoV-2 S plasmid was a kind gift from the Krammer laboratory at Icahn School of Medicine, Mount Sinai. The spike gene was amplified from the plasmid and subcloned in the BacMam vector with a C-terminal HRV 3C cleavage tag followed by a seven-histidine and twin Strep tag. Bacmid DNA and virus were prepared as described in the Invitrogen Bac-to-Bac manual. After two generations of amplification in Sf9 cells, the V2 virus was used for transfection of HEK293F cells at a density of 2 million per millilitre. Sodium butyrate (4 m*M*) was added to enhance the production of protein 8 h post-infection. The medium supernatant containing the secreted spike protein was harvested on day 3 by centrifuging the cells at 150*g* for 10 min. The medium was incubated with pre-equilibrated Ni–NTA (Qiagen) beads at room temperature for 1–2 h (1 ml of beads per 200 ml of medium). The Ni–NTA beads were washed with phosphate-buffered saline (PBS) containing 20 m*M* imidazole, followed by elution with 280 m*M* imidazole in PBS. The eluted protein was run on SDS–PAGE to assess its purity, further concentrated and injected onto a 24 ml Superdex 200 (Cytiva) size-exclusion column to exchange the buffer to 50 m*M* Tris pH 8, 200 m*M* NaCl, 1 m*M* DTT. To cleave the tag, the eluted fractions from Ni–NTA chromatography were diluted with 50 m*M* Tris pH 8, 200 m*M* NaCl, 1 m*M* DTT and incubated with HRV 3C protease overnight at 4°C, followed by reverse IMAC to obtain the spike protein without tag in the flowthrough. The flowthrough was concentrated using an Amicon 100 kDa concentrator, flash-frozen using liquid nitrogen and stored at −80°C until further use.

β-Galactosidase with an N-terminal 6×histidine tag followed by a thrombin cleavage tag was a gift from Professor Doug Juers; it was cloned in the pET-15b vector and transformed in *Escherichia coli* strain JM109 (Juers *et al.*, 2000 ▸). The cells from a glycerol stock were patched onto LB agar with ampicillin and allowed to grow overnight at 37°C. A single colony was picked and allowed to grow in lysogeny broth (LB) with ampicillin overnight. The next day, 4 l LB with 100 µg ml^−1^ ampicillin was inoculated and the OD_600_ was monitored every 30 min. The expression of protein was induced by adding 1 m*M* isopropyl β-d-1-thiogalactopyranoside when the OD of the cells reached 0.6, and the cells were allowed to grow for 4–5 h at 37°C. Subsequently, the cells were harvested by centrifugation at 4000*g* for 20 min and the cell pellet was flash-frozen and stored at −80°C until further use. On the day of purification, the cells were resuspended in ∼50 ml resuspension buffer consisting of 20 m*M* Tris pH 8, 500 m*M* NaCl, 5 m*M* imidazole, 1 m*M* β-mercaptoethanol (β-ME), 1 m*M* MgCl_2_ and sonicated for 5 min (5 s on, 10 s off) at 40% amplitude. The lysate was clarified by centrifugation at 18 000*g* for 45 min and the supernatant was collected. A 5 ml Ni–NTA column was equilibrated with 5 column volumes (CV) of resuspension buffer, followed by application of the supernatant at a flow rate of 1 ml min^−1^. After binding of the protein, the column was washed with 50 CV of resuspension buffer followed by a linear gradient elution with buffer *B* (resuspension buffer with 500 m*M* imidazole); 1.8 ml fractions were collected. The fractions from Ni–NTA elution were analysed by SDS–PAGE and the fractions from the last half of the peak were pooled and dialyzed with 2 × 4 l dialysis buffer consisting of 25 m*M* Tris pH 8, 125 m*M* NaCl, 2.5 m*M* CaCl_2_, 2 m*M* β-ME. The protein was concentrated using a 100 kDa cutoff Amicon concentrator, aliquoted and stored at −80°C. An aliquot of this protein was thawed and injected into a Superdex 200 column, and the buffer was exchanged to 100 m*M* Tris pH 8, 200 m*M* NaCl, 2.5 m*M* MgCl_2_, 5 m*M* CaCl_2_, 2 m*M* β-ME. Cleavage of the tag was carried out by thrombin protease at room temperature for 4 h, followed by reverse IMAC to collect the protein without tag in the flowthrough. The protein without tag was concentrated and grids were prepared.

### 2.2. Grid preparation

6.3 µl of the protein was thawed on ice and 0.7 µl of 10× additive (surfactant) stock was added to obtain a final concentration of 1×. This sample was incubated on ice for 2–5 min and then centrifuged at 21 000*g* for 20 min. Meanwhile, a Vitrobot Mark IV (Thermo Fisher Scientific) chamber was equilibrated at 20°C (unless stated otherwise) and 100% humidity. Quantifoil 1.2/1.3 or Quantifoil 0.6/1 grids were glow-discharged in a reduced-air environment with a PELCO easiGlow chamber using a standard setting of 25 mA current for 1 min. The grid was mounted on the Vitrobot Mark IV and 3 µl of sample was applied to the grid. A blotting time of 3–4 s, a wait time of 10 s and a blot force of 0 were used to obtain a thin film of the specimen. For data sets where grids were prepared at different temperatures, the protein was incubated on a thermal block at the required temperature for 3–7 min before applying it to the grid. The Vitrobot chamber was maintained at the required temperature and 100% humidity. The blot time, blot force and wait time were kept constant.

### 2.3. Grid screening and data collection

Grids were screened on a Titan Krios microscope operating at 300 kV using standard low-dose settings, and automated data collection was set up either on a Falcon 3 or Gatan K2 detector in counting mode with the *EPU* software (Thermo Fisher Scientific). A magnification of 59 000× was only used for the catalase 37°C data set, with a pixel size of 1.38 Å (and for PaaZ incubated at 37°C with no additive, but no data were collected). For all of the other data sets collected on the Falcon 3 dectector, a magnification of 75 000×, corresponding to a pixel size of 1.07 Å, and a dose of approximately 1 e^−^ Å^−2^ per frame were used and movies of 24–25 frames were collected. The data sets collected on the K2 detector (Gatan) were collected in EFTEM mode with a slit width of 20 eV and a nominal magnification of 130 000×, corresponding to a pixel size of 1.08 Å. The dose was 5 e^−^ per pixel per second and an exposure of 8 s was used. Each movie was fractionated into 32 frames with a total dose of ∼36.28 e^−^ Å^−2^. The grid-preparation conditions and data parameters are summarized in Table 1 ▸.

### 2.4. Data processing and model refinement

*RELION* 3.1 (Scheres, 2012 ▸; Zivanov *et al.*, 2018 ▸) was used to process all of the data using a standard workflow, which is summarized as follows. The multi-frame movies were summed and corrected for beam-induced motion using the inbuilt *MotionCorr* algorithm in *RELION*. The resulting summed micrograph images were used to estimate the contrast transfer function (CTF) using *GCTF* (Zhang, 2016 ▸). The micrographs after CTF correction were used to pick particles using either reference-free methods such as Laplacian-of-Gaussian (LoG)-based picking in *RELION* or *Gautomatch* (https://www2.mrc-lmb.cam.ac.uk/download/gautomatch-053/) or using previously obtained 2D templates. After picking, the particles were boxed and extracted using box sizes of 256 or 320 pixels (Table 1 ▸). These particles were then subjected to multiple rounds of iterative 2D classification to obtain good-quality, high-resolution classes. Either a low-resolution initial model generated from the same data set or a low-pass filtered model from a pre-existing data set was used as a reference for 3D refinement. The selected good-quality particles were then used for either 3D classification or 3D refinement based on the data (Scheres, 2012 ▸). If the resolution at this stage was below 4.5 Å, CTF refinement and Bayesian polishing were performed on the final particles to correct for beam tilt, anisotropic magnification, per-particle defocus and the effects of beam-induced motion, respectively (Zivanov *et al.*, 2018 ▸, 2019 ▸). Another round of 3D refinement was performed after this step. Further, postprocessing was performed to sharpen the final map and estimate the final global resolution (Rosenthal & Henderson, 2003 ▸).

The PaaZ–cetyltrimethylammonium bromide (CTAB) and spike–CTAB data sets were subjected to multiple rounds of 3D classification. For most data sets (with the exception being the CRP pentamer), as a part of the standard workflow the resulting map after reconstruction had the wrong hand and the maps were flipped to obtain the correct hand before the model was fitted and refinement was performed. The 3*DFSC* server was used to calculate the sphericity of the final maps (Tan *et al.*, 2017 ▸). The *E*_od_ was calculated with *cryoEF* using a subset of 1000 angles and the appropriate symmetries for the respective molecules (Naydenova & Russo, 2017 ▸). *ChimeraX* (Pettersen *et al.*, 2004 ▸) was used to generate electrostatic potential maps of the structures. The *PyMOLAPBS* plugin (Schrödinger) was used to generate the electrostatic potential map for PaaZ. *EMAN*2 (Tang *et al.*, 2007 ▸) and *Phenix* (Liebschner *et al.*, 2019 ▸) were used to generate the map versus model FSCs. The final model refinement was performed in real space for the PaaZ and CRP data sets using *Phenix* (Afonine *et al.*, 2018 ▸) and in reciprocal space using *REFMACServalcat* (Murshudov *et al.*, 2011 ▸; Yamashita *et al.*, 2021 ▸) for the catalase and spike data sets. Initial water picking in the PaaZ data set was performed with *Coot* (Emsley *et al.*, 2010 ▸) and was manually inspected to remove waters that were not hydrogen-bonded to amino-acid residues, also using the *F*_o_ − *F*_c_ omit map from *Servalcat* (Yamashita *et al.*, 2021 ▸); a conservative approach was used so that noise was not modelled. Figures were generated with *Chimera* and *PyMOL* (Pettersen *et al.*, 2004 ▸; Schrödinger).

## 3. Results

### 3.1. Analysis of preferred views of selected macromolecules

**Figure 2:**
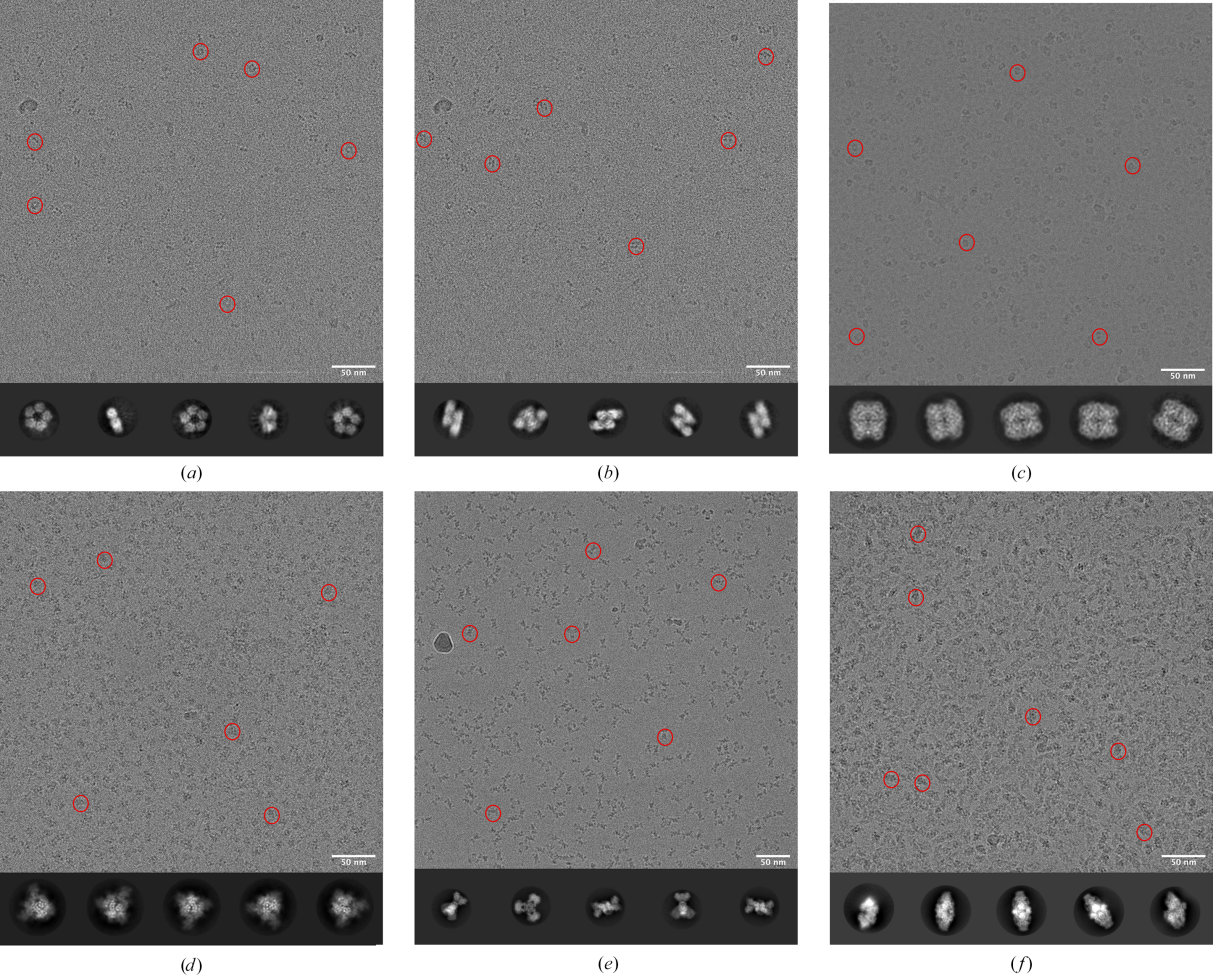
Representative micrographs, with a few selected particles indicated with red circles, and 2D class averages of the test proteins used in this study. (*a*) The C-reactive protein (CRP) pentamer adopts a preferred bottom view, which shows the pentameric arrangement of the monomers. (*b*) The CRP decamer adopts a preferred side view, which shows the staggered arrangement of two CRP pentamers stacked on top of each other. The same micrograph is used in (*a*) and (*b*). (*c*) Catalase adopts a preferred top view, as seen in the micrograph and 2D class averages. (*d*) SARS-CoV-2 spike adopts a preferred bottom view showing the trimeric arrangement. (*e*) PaaZ adopts a preferred side view, as seen in the 2D class averages, and the micrograph shows occasional clumping of hexamers on the grids. (*f*) β-Galactosidase with an N-terminal polyhistidine tag adopts a preferred side view, as seen in the 2D class averages, and the micrograph shows aggregation on grids. For the above data sets, the catalase and PaaZ grids were prepared at 4°C and all other grids were prepared at 20°C.

A set of well characterized proteins with varying molecular weights, shapes, symmetries and surface-charge distributions and with known experimental structures were selected to rule out any artefacts that may arise owing to changes in the conditions of sample preparation. The test proteins used in the analysis include human C-reactive protein (CRP) pentamer, CRP decamer, human erythrocyte catalase, SARS-CoV-2 spike protein, *E. coli* PaaZ and *E. coli* β-galactosidase. Micrographs and 2D class averages of these data sets collected at the start of the study are shown in Fig. 2 ▸.

CRP exists in a concentration-dependent equilibrium between a pentamer and a decamer of a 25 kDa polypeptide under physiological conditions (Okemefuna *et al.*, 2010 ▸). In the concentration range used for cryo-EM experiments, both pentamer and decamer populations were observed in the same micrograph and were analysed separately (Figs. 2 ▸*a* and 2 ▸*b*). The effect that these populations may have on each other’s behaviour in the thin film is an interesting concept that has not been analysed in the current study but is discussed briefly. *C*5 symmetry was applied to the smaller pentameric molecule and no symmetry was applied to the decamer during image processing. The preferred view for the CRP pentamer is the top/bottom view, where the symmetric arrangement of the monomers can be seen. Some side views are observed, but no tilted side views are seen in the 2D class averages (Fig. 2 ▸*a*). In contrast, in the case of the CRP decamer side views are predominantly observed and no top/bottom views are seen (Fig. 2 ▸*b*). Catalase exists as a dimer of tetramers and possesses *D*2 symmetry (Ko *et al.*, 2000 ▸). It adopts a preferred top/bottom view on the grids when the grids are prepared at 4°C using Quantifoil 0.6/1 holey carbon grids (Fig. 2 ▸*c*). This enzyme has previously been noted for its peculiar behaviour during cryo-EM grid preparation and has been used as a test specimen to overcome orientation bias, and we used it as a control sample and to probe additional parameters (Chen *et al.*, 2022 ▸; Fan *et al.*, 2021 ▸; Vinothkumar & Henderson, 2016 ▸). The SARS-CoV-2 spike protein is now a well studied trimeric viral membrane protein whose symmetry can vary depending on the conformation of the receptor-binding domain (Huang *et al.*, 2020 ▸; Wrapp *et al.*, 2020 ▸). The spike trimer has three receptor-binding domains (RBDs), one per monomer, and they can adopt different conformational states and thus dictate the symmetry of the protein. Soluble spike protein shows a preference for a bottom view on grids in the absence of an additive (Fig. 2 ▸*d*). In this data set, we observed that there was heterogeneity in the RBD domain, and hence *C*1 symmetry was applied for the rest of the analysis.

PaaZ is a bifunctional enzyme and consists of six poly­peptides, where a domain-swapped dimer assembles to form a trimeric structure. In an earlier study, when frozen in ice, the enzyme was found to form clumps and aggregates, which was overcome by the use of graphene oxide and a low concentration of protein (Sathyanarayanan *et al.*, 2019 ▸). PaaZ adopts a preferred side view in the absence of an additive when frozen in ice at 4°C, with a tendency to clump or cluster (Fig. 2 ▸*e*). Nevertheless, the isolated particles were sufficient to obtain reasonable maps, and here we were interested in screening additional parameters to check whether some of the aggregation and clumping could be overcome to enhance the quality of the data for the enzyme in ice. β-Galactosidase from *E. coli* is a well studied enzyme as well as a test specimen in cryo-EM (Bartesaghi *et al.*, 2018 ▸; Juers *et al.*, 2012 ▸). It is a glycoside hydrolase enzyme, similar to catalase, and exists as a tetramer with *D*2 symmetry. When purified with a tag at its N-terminus, the enzyme adopts a preferred side view and tends to form clumps or aggregates in ice (Fig. 2 ▸*f*). Of the standard proteins tested, spike, PaaZ and β-galactosidase were obtained by overexpression and had additional polyhistidine affinity tags at either the N- or C-termini of their monomers.

### 3.2. Surfactants affect macromolecule orientation distributions in a charge-dependent manner

We tested a specific set of surface-active molecules with varying head-group charges (cationic, anionic and non-ionic) and chain-length packing (unsaturated versus saturated alkyl chains), differing in their critical micelle concentration (CMC) and concentration, on the set of proteins described above. All of the surfactants were used at concentrations lower than their respective CMC (except Tween 80 and Amphipol A8-35). The properties of the surfactants are listed in Table 2 ▸.

**Figure 3:**
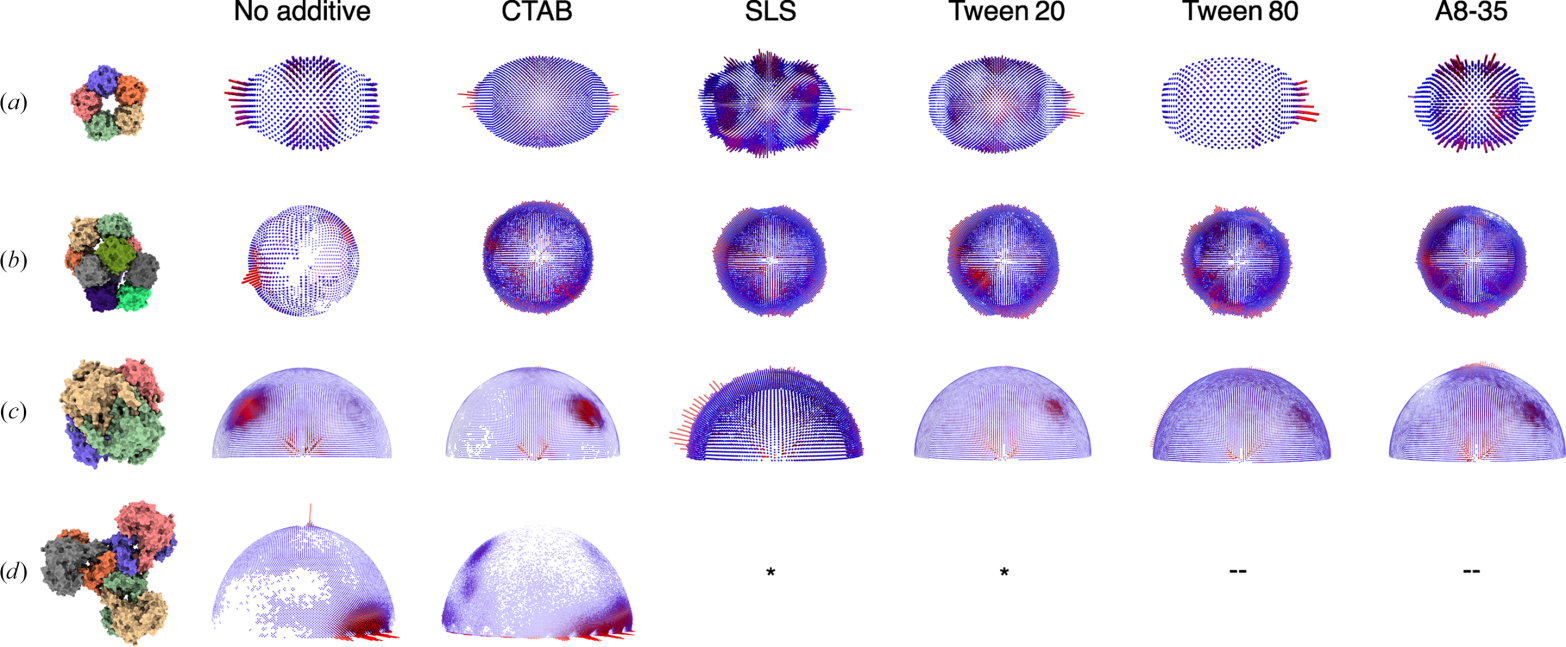
Orientation-distribution plots from *RELION* (Scheres, 2012 ▸) of proteins upon the addition of surfactants with varying properties to the sample buffer before grid preparation. The reference structures of the respective proteins are generated by creating a surface representation in *ChimeraX* from models from PDB entries 7pkb, 7pk9, 1dgf and 6jql. (*a*) Changes in the CRP pentamer orientation distribution upon the addition of surfactants. The distributions are distinct from each other, except for Tween 20 and Tween 80, which have similar distributions. (*b*) Changes in the CRP decamer orientation distribution upon surfactant addition; all surfactants lead to a similar even orientation distribution. (*c*) Changes in the catalase orientation distribution upon the addition of surfactants, where the charged surfactants have distinct distributions (CTAB and SLS) and the neutral surfactants (Tween 20 and Tween 80) and A8-35 show similar distributions. (*d*) Changes in PaaZ orientation distributions upon the addition of the cationic surfactant CTAB. The effects of SLS and Tween 20 on PaaZ were also tested, but visual inspection of the micrographs (Supplementary Fig. S3) showed no improvement and no data were collected; therefore they are not included (marked by asterisks). The effects of Tween 80 and A8-35 on PaaZ were not tested.

Upon addition of the cationic surfactant cetyltrimethyl­ammonium bromide (CTAB), a change in the orientation distribution was observed for all of the tested samples (Fig. 3 ▸). An improvement in the orientation distribution of CRP pentamer and decamer was observed, resulting in isotropic 3D reconstructions (Figs. 3 ▸*a* and 3 ▸*b* and Table 3 ▸). For catalase, the initial no-additive data set was collected from a specimen prepared at 4°C on a Quantifoil 0.6/1 grid, which has a strong preference for the top/bottom views (Fig. 2 ▸*c*). However, as the grids for all of the additive data sets were made at 20°C, for an appropriate comparison we also prepared catalase grids at 20°C with no additive. This data set shows a preference for a tilted side view, annotated as side view A (Supplementary Fig. S2). The changes in the orientation distributions of catalase are a result of variations in the incubation temperature and the grid hole geometry, which we did not anticipate to have such a large effect on the quality of the reconstruction and were explored further in this study (discussed below). Upon the addition of CTAB to catalase grids, the preference for the tilted side view A was lost, and the particles instead showed a preference for the other tilted side view B, and a map with similar quality to the no-additive data set was obtained (Fig. 3 ▸*c* and Table 3 ▸). In the case of PaaZ, the preference for the side view was maintained even in the presence of CTAB, but additional side-tilted views were sampled, which led to an improved resolution and a higher quality map (Fig. 3 ▸*d* and Table 3 ▸). The addition of a negatively charged detergent, sodium lauryl sarcosine (SLS), resulted in evenly distributed orientation distributions, with no preference for any particular view, for all cases tested except PaaZ (Fig. 3 ▸). The resulting maps with SLS as an additive are of lower resolution in all cases, which may be due to a lower number of particles or to the ice thickness and a possible effect of the anionic head group (Table 3 ▸).

The addition of the non-ionic detergents Tween 20 and Tween 80 to CRP pentamer, CRP decamer and catalase led to evenly distributed orientations (Fig. 3 ▸), but PaaZ showed aggregation on grids (Supplementary Fig. S3). Tween 80 was tested as it is known to form denser layers at the AWI compared with Tween 20 due to differences in the alkyl chain (length and unsaturation; Szymczyk *et al.*, 2018 ▸). Additionally, it has been shown to differ in its ability to separate protein films from the AWI compared with Tween 20 (Rabe *et al.*, 2020 ▸). For the macromolecules tested in our study, no significant difference was observed between the orientation distributions obtained from either of the Tween surfactant data sets (Fig. 3 ▸). However, we note that with CRP pentamer, the orientation distributions look similar with both Tween 20 and Tween 80, but only the addition of Tween 20 led to an isotropic map (*R* = 3.3 Å), while the addition of Tween 80 led to a low-resolution map (*R* = 7.5 Å). These differences may be a result of variations in ice thickness or due to differential interaction of Tween 80 with CRP pentamer, and further experiments need to be performed to determine the cause of this discrepancy. Amphipol A8-35, a surface-active ionic polymer that does not form typical micelles and thus differs from the other surfactants, was also tested to observe its effect on orientations. The orientation distributions obtained in this case were similar to those of the Tween 20 and Tween 80 data sets (Fig. 3 ▸). The observed results with the given test samples indicate that the charge on the detergent head group is an important parameter, whereas the chain length and saturation of the hydrophobic chain have very little effect in modulating the orientation distributions of the macromolecules.

To understand the importance of the discrete views that were sampled and their contribution to the quality of the map, the efficiency of the orientation distribution (*E*_od_) was calculated using *cryoEF* (Naydenova & Russo, 2017 ▸) and the sphericities of the final maps were calculated using the 3*DFSC* server (Tan *et al.*, 2017 ▸; Table 3 ▸). *E*_od_ provides an estimate of the coverage of the 3D Fourier space from the 2D projections in the data, whereas sphericity provides a measure of anisotropy in the final maps by estimating the directional resolution. Among the data sets, the CTAB data sets stand out and result in higher resolution maps. We think that this could be a result of optimum ice thickness, along with a sampling of the orientations that contribute more to the reconstruction, which is also demonstrated in Supplementary Fig. S1 by using a subset of particles from the catalase data as an example.

### 3.3. The presence of a solvent-exposed polyhistidine tag affects protein orientations in thin films

**Figure 4:**
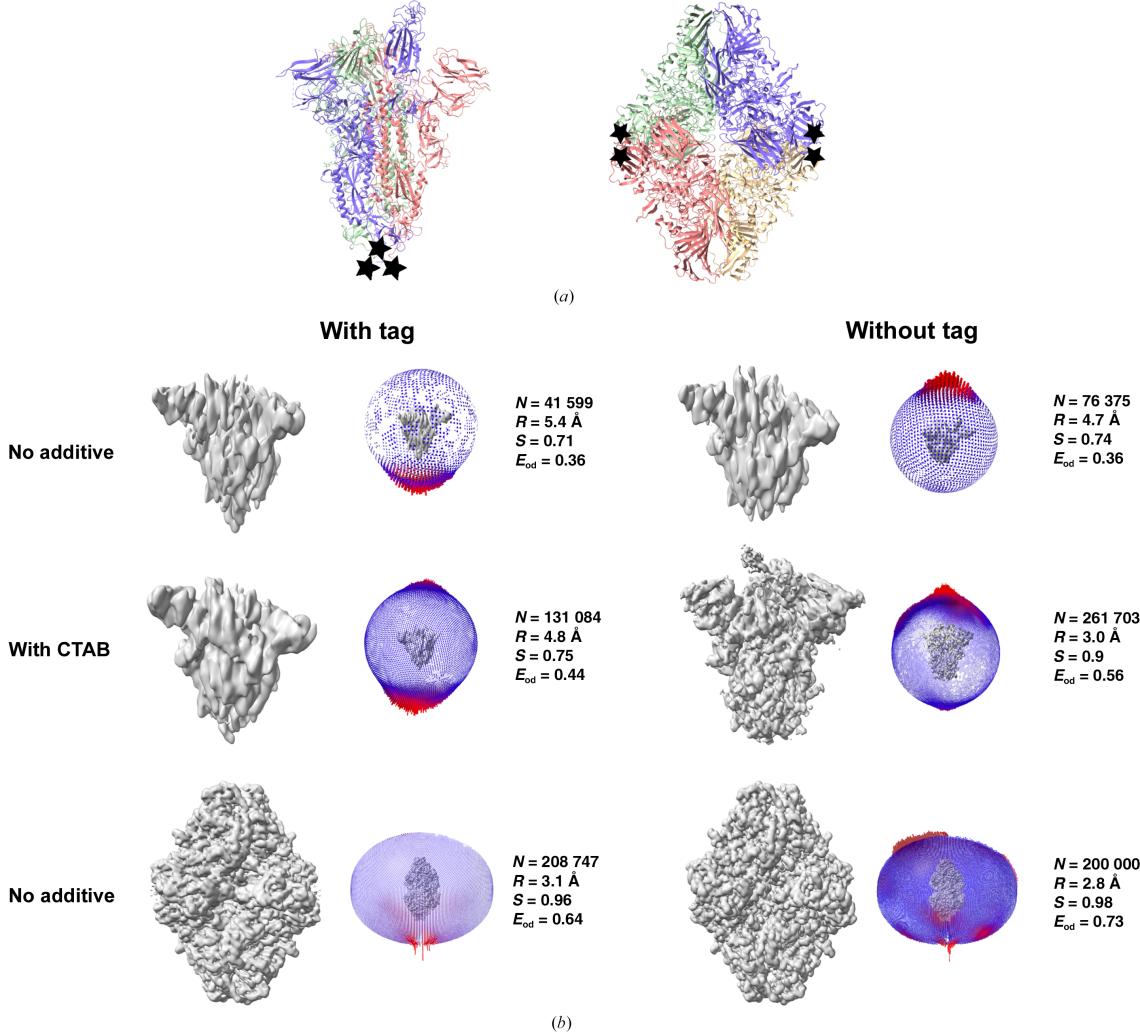
The effect of a polyhistidine affinity tag on the SARS-CoV-2 spike protein and β-galactosidase orientation distributions. The different parameters that are used to analyse the quality of the maps are shown next to the orientation plots. *N* indicates the number of particles used for reconstruction, *R* indicates the final resolution of the map, *S* indicates the sphericity and *E*_od_ indicates the efficiency of Fourier space coverage. (*a*) The locations of the tags on the protein models are indicated by black stars. The models used as references are PDB entries 8h3d and 6cvm for the spike protein and β-galactosidase, respectively. (*b*) The orientation-distribution plots of the spike protein change upon removal of the affinity tag, but the change is not sufficient to obtain an isotropic map. The addition of the cationic surfactant CTAB further alters the orientations of the spike protein without tag and leads to a more isotropic map. β-Galactosidase enzyme (bottom panel) orientations change upon removal of the affinity tag and lead to an isotropic high-resolution map without any additive. The unsharpened final combined maps are shown in grey in (*b*).

SARS-CoV-2 spike protein and *E. coli* β-galactosidase are two samples that have been well studied by cryo-EM, and multiple structures have been reported in the past (Bartesaghi *et al.*, 2018 ▸; Bodakuntla *et al.*, 2023 ▸; Esfahani *et al.*, 2024 ▸; Hardenbrook & Zhang, 2022 ▸; Harvey *et al.*, 2021 ▸; Juers *et al.*, 2012 ▸; Wrobel, 2023 ▸). However, we observed orientation bias for these two recombinantly purified proteins, with differing severity. The spike protein showed severe orientation bias, which resulted in anisotropic maps of poor quality that could not be improved by surfactant addition (data not shown). β-Galactosidase showed comparatively less orientation bias and led to a reconstruction of moderate quality, but the map was still anisotropic (Fig. 4 ▸*b*). Given that both of these proteins feature polyhistidine affinity tags, and considering that the preferred view in both cases may involve an exposed tag, we hypothesized that this tag could be inducing a bias in orientation (Fig. 4 ▸*a*).

Thus, the affinity tags were removed from the spike protein and β-galactosidase by protease cleavage, which led to a change in the orientation distributions in both cases (Fig. 4 ▸). In the case of spike, although the orientation bias remained, the preference shifted from the bottom view (where the histidine tag is attached) to the top view (Fig. 4 ▸*b*). Subsequently, CTAB was added to the sample, imaging was performed on thin ice and side-tilted views were obtained, which resulted in a reasonable isotropic map. The final reconstruction was obtained with 261 703 particles to an overall resolution of 3 Å (Fig. 4 ▸*b*). At this resolution, amino-acid side chains and glycosylation on the protein surface were visible in the ordered regions, but the regions of the RBD were poorly resolved, as substantiated by the local resolution plot (Supplementary Fig. S4). Further, we used the spike data set to evaluate the effect of different post-processing methods and the fit of the model to the map (He *et al.*, 2023 ▸; Pintilie *et al.*, 2020 ▸; Sanchez-Garcia *et al.*, 2021 ▸; summarized in Supplementary Fig. S4). In comparison to the other data sets, an increase in *E*_od_ and sphericity is evident in this case (Fig. 4 ▸*b*). To verify that the improvement in map quality is a result of new orientations and not an increase in the total number of particles, all of the top/bottom views were removed for 3D refinement and a reconstruction using only the side/tilted views corresponding to ∼94 000 particles was performed. This resulted in a map with a sphericity of 0.88 and a resolution of 3.4 Å (Supplementary Fig. S5), similar to that with all of the particles, illustrating that the top/bottom views contribute little information to the final reconstruction of spike protein. In the case of β-galactosidase, the orientation bias was reduced and alternate views were sampled, as indicated by the orientation plot and an improvement of the *E*_od_ from 0.64 to 0.73 when the tag was cleaved. The resulting reconstruction also shows improvement, as indicated by visual inspection of the map (Fig. 4 ▸*b* and Supplementary Fig. S6) and the improvement in resolution and sphericity. Enlarged areas of the map to highlight the difference in map quality and comparison of the half map and model versus map FSCs of the β-galactosidase are shown in Supplementary Fig. S6.

### 3.4. The temperature of the incubation chamber during freezing affects protein orientations

**Figure 5:**
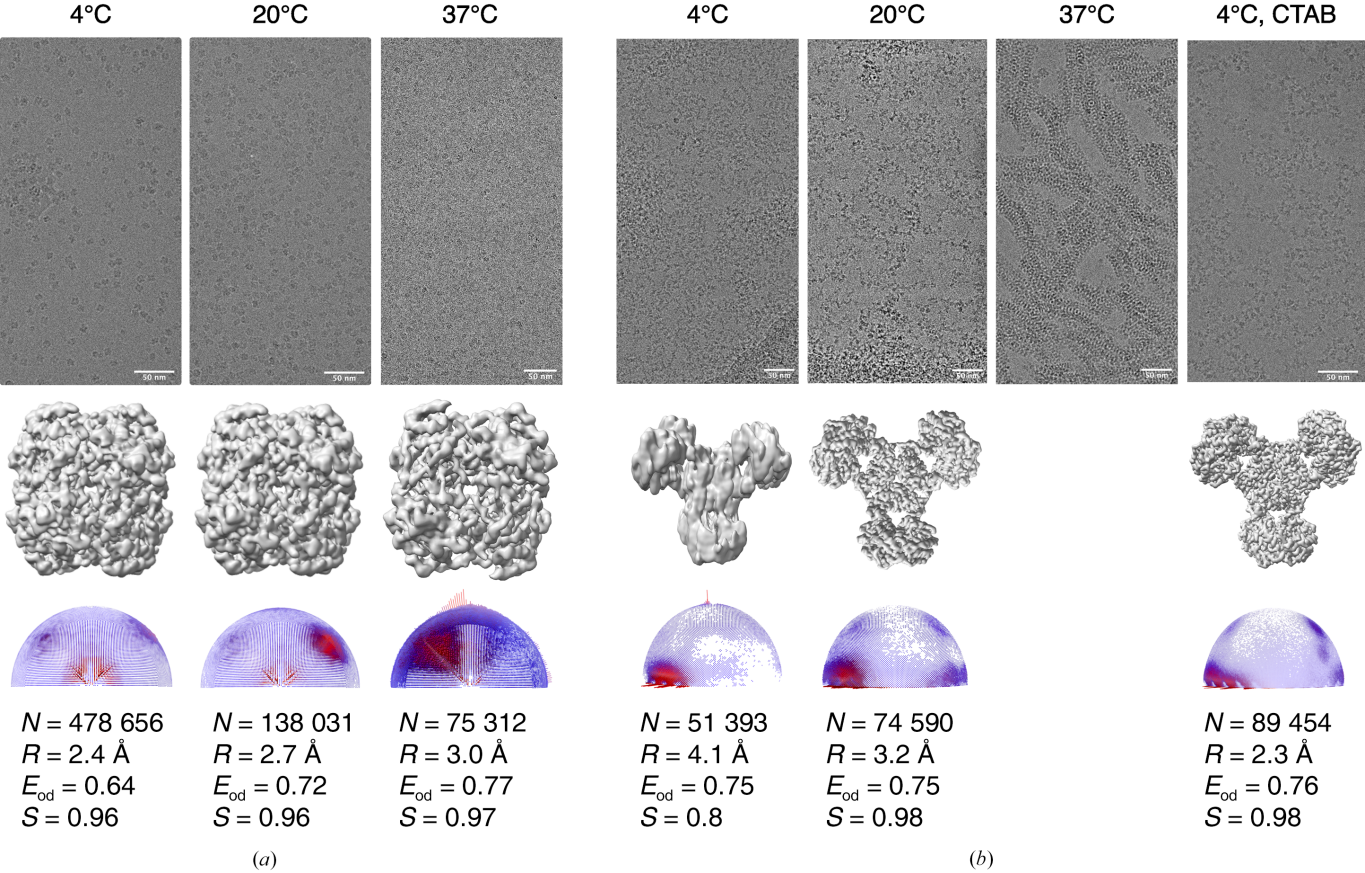
The effect of temperature during cryo-EM sample preparation of catalase and PaaZ. Micrographs, maps, orientation-distribution plots and the different parameters that are used to analyse the quality of the maps are shown. *N* indicates the number of particles used for reconstruction, *R* indicates the final resolution of the map, *S* indicates the sphericity and *E*_od_ indicates the efficiency of Fourier space coverage. (*a*) Catalase orientation distributions change significantly when grids are blotted at different temperatures in the absence of any additive. (*b*) PaaZ orientation distributions change slightly when grids are held and blotted at different temperatures in the absence of any additive. In the case of PaaZ, the condition with grids prepared at 4°C with CTAB as an additive is included for comparison as this combination led to a high-resolution isotropic map.

The temperature at which the grids are held and blotted is critical for determining ice thickness, protein stability and dynamics. Hence, grids were prepared at different temperatures to determine the effect of temperature on the orientation distributions. Typically, lower temperatures are used during sample application to grids to keep the protein stable (except in some cases, such as microtubules, where a higher temperature is a prerequisite), and temperature is not very often screened as a parameter to overcome preferred orientations. For catalase, the orientation distributions varied significantly when grids were prepared at different temperatures. The coverage of the 3D Fourier space (*E*_od_) improved from 0.64 at 4°C to 0.72 at 20°C and 0.77 at 37°C (Fig. 5 ▸*a*). However, the resolution of the final reconstruction was best for the 4°C data set, which could be due to the higher number of particles used for reconstruction; that is, 478 656 compared with 138 031 for the 20°C data set and 75 312 for the 37°C data set. The respective *B* factors (from *RELION* post-processing) for the reconstructions were −107, −110 and −126 Å^2^.

In the case of PaaZ, when grids were prepared at 4, 20 and 37°C increasing higher order structures were observed, particularly at 37°C. At 4°C higher order structures were minimal, but a preference for the side view was observed. Data collected at 4 and 20°C have a similar *E*_od_, and the orientation-distribution plots show a slight increase in the sampling of additional side-tilted views at 20°C, as indicated by a reduction in empty spaces in the orientation plots obtained from *RELION* (Fig. 5 ▸*b*). However, clumping of the particles was still observed at 20°C. Hence, CTAB was added to the sample and the grids were prepared at 4°C, which led to less clumping and consequently resulted in a high-resolution map at 2.3 Å, as described further in the next section.

It is evident from these observations that physical factors, such as the grid-preparation temperature, can affect protein behaviour and should be considered as an important screening condition when dealing with orientation bias along with surfactants.

### 3.5. High-resolution map of *E. coli* PaaZ in ice

**Figure 6:**
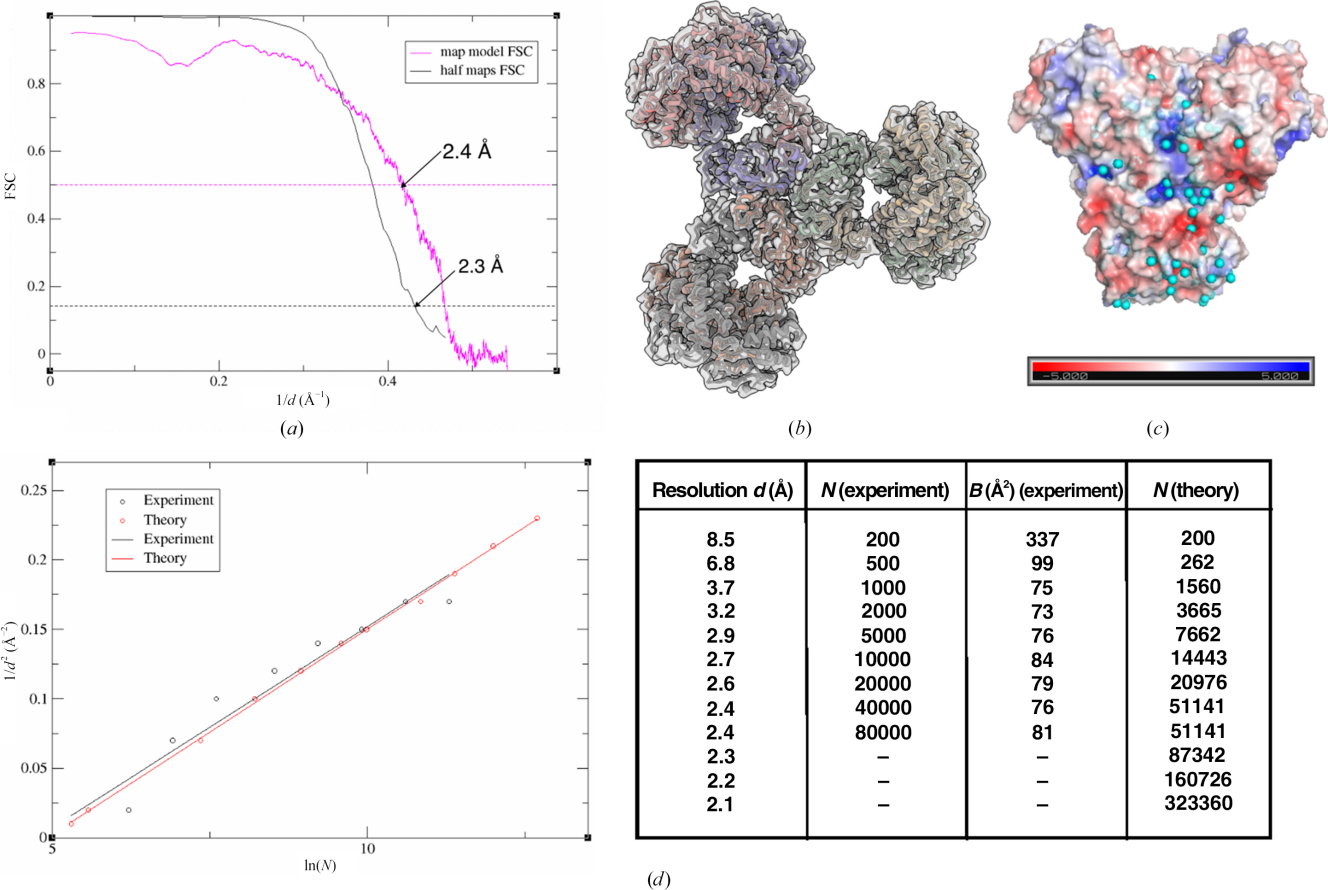
High-resolution cryo-EM map from PaaZ grids prepared at 4°C with CTAB additive. (*a*) Comparison of the half-maps and map-versus-model FSCs of the PaaZ data set. (*b*) The six polypeptides coloured individually and in cartoon representation fitted into the cryo-EM map (transparent grey) of PaaZ. (*c*) Electrostatic potential surface representation of the domain-swapped PaaZ dimer with waters modelled and shown as cyan spheres. (*d*) ResLog plot of PaaZ with the experimental and theoretical numbers of particles required to reach a particular resolution. *N* indicates the number of particles used for reconstruction, *d* is the resolution and *B* indicates the *B* factor, as estimated by *RELION* post-processing. *D*3 symmetry was applied for the reconstruction and the ResLog plot indicates the number of particles used, not the number of asymmetric units averaged.

In order to understand the mechanism of action of a macromolecule, high-resolution maps are required to build an accurate model and, more importantly, to model the solvent molecules and other ligands of interest. We previously reported the structure of PaaZ, a bifunctional enzyme from *E. coli* that catalyzes two steps of the phenylacetic acid degradation (paa) pathway. In the previous study, the structure of PaaZ was determined at ∼2.9 Å resolution using graphene-oxide support grids (Sathyanarayanan *et al.*, 2019 ▸). During this study, it was observed that the enzyme tended to clump when frozen in ice in the absence of any additive, and the use of a graphene oxide support and a low concentration of enzyme yielded a very good distribution of particles. In the current study, we obtained a high-resolution map using CTAB as an additive during grid preparation, with *D*3 symmetry and higher order aberration correction during image processing. The resolution estimated by comparison of the half-map FSC (at a 0.143 threshold) is 2.3 Å, and a comparable resolution of 2.4 Å is obtained from comparison of the map-versus-model FSC (at a 0.5 threshold; Fig. 6 ▸*a*). The model-fitted PaaZ structure is shown in Fig. 6 ▸(*b*). Several water molecules could be modelled at this resolution and an electrostatic potential map of the dimer with water molecules modelled as spheres and coloured cyan is shown in Fig. 6 ▸(*c*). To further analyse the quality of the data, the ResLog plot was calculated by obtaining a 3D reconstruction using different numbers of particles and plotting the resolution (1/*d*^2^) obtained against the number of particles (ln*N*) used (Fig. 6 ▸*d*; Rosenthal & Henderson, 2003 ▸). From the experimental data, it can be seen that as the resolution approaches the Nyquist limit of 2.1 Å, the addition of more particles (from 40 000 to 80 000) does not lead to an improvement in resolution. The slope of this graph was used to calculate a *B* factor of 69.4 Å^2^, which is slightly lower than the *B* factor estimated by the post-processing option in *RELION*, which was used for map sharpening (−75 Å^2^). A 200-particle data set was used as a base, and the particles required to obtain a certain resolution were calculated theoretically. These theoretical estimates are in agreement with the experimental values.

## 4. Discussion

In this era of artificial intelligence-based protein structure prediction (Baek *et al.*, 2021 ▸; Jumper *et al.*, 2021 ▸; Schauperl & Denny, 2022 ▸), there is still no substitute for the joy one feels when the molecule of interest is imaged and observed for the first time after the hard work of expression and purification. However, the first data collection for a new sample often does not result in a high-resolution structure, as there can be one or more challenges that need to be overcome. Of these, the preferred orientation of macromolecules and the possibility of denaturation due to exposure to the AWI is a serious issue and is one of the major bottlenecks. It is now known that the tendency of macromolecules to adopt a preferential orientation when frozen in ice using cryo-EM grids is a consequence of protein–AWI interaction (Chen *et al.*, 2019 ▸; Noble, Wei *et al.*, 2018 ▸), but the nature of this interaction remains unclear. On the other hand, amphipathic molecules have been used in cryo-EM grid preparation since the 1980s (Frederik *et al.*, 1989 ▸). Their use in solving the preferred orientation of proteins by modulating the AWI has recently been appreciated in cryo-EM and is now being used more routinely for high-resolution structure determination (Chen *et al.*, 2019 ▸, 2022 ▸; Li *et al.*, 2021 ▸). At a fundamental level, several open questions remain. What causes proteins to adopt certain preferred views? How do surfactants affect this behaviour? What are the roles of other physical properties such as ice thickness, surface tension and temperature in the behaviour of macromolecules during freezing? To address some of these questions, we systematically tested grid-freezing conditions on a set of well characterized proteins that adopt preferred orientations. The selected proteins had molecular weights ranging from 125 to 466 kDa and varying symmetries (Fig. 2 ▸). The degree of orientation bias also varied significantly. The CRP decamer and SARS-CoV-2 spike had a more severe orientation bias, with more than 90% abundance of the preferred view, whereas in the case of the CRP pentamer the abundance was 50% and in catalase it was near 60%. Additionally, spike, PaaZ and β-galactosidase were recombinantly overexpressed and had a polyhistidine affinity tag at the termini of each monomer, whereas CRP and catalase were purified from native sources and did not contain any affinity tags.

The AWI exhibits an affinity for OH^−^ ions from water, resulting in a negative charge (Chaplin, 2009 ▸; Drzymala *et al.*, 1999 ▸). Extensive research has been conducted on the behaviour of proteins and surfactants at the AWI and in thin films, spanning the fields of cryo-EM and surface chemistry (Li *et al.*, 2021 ▸; Rabe *et al.*, 2020 ▸; Samanta & Ghosh, 2011 ▸; Szymczyk *et al.*, 2018 ▸). Based on these investigations and an examination of the surface-charge distributions of macromolecules in our study, we postulated that electrostatic interactions may play a pivotal role in dictating protein orientations. To visualize the surface-charge distributions of the proteins, electrostatic potential maps were calculated by assigning charges using the *PDB*2*PQR* server (Jurrus *et al.*, 2018 ▸) at a given pH and were visualized using *ChimeraX* (Pettersen *et al.*, 2021 ▸). It was observed that the charge on the preferred view was positive in the case of catalase, neutral in CRP pentamer, CRP decamer and PaaZ, and negative and neutral in β-galactosidase (Supplementary Fig. S7). The preferred bottom view of the spike is negatively charged, but this charge is likely to be masked by the presence of a 56-amino-acid affinity tag in each monomer. Further, the charge on the alternate view of β-galactosidase with tag, which is not seen in the 2D classes, is more negative compared with the preferred view (Supplementary Fig. S7*c*).

An interesting sample in our study is C-reactive protein (CRP), which exists as both pentamer and decamer populations dependent on concentration, and the impact of these populations on each other’s orientation distribution is intriguing. The particle numbers of each population of CRP and their relative abundance on the micrographs (after 2D classification) are summarized in Supplementary Table S1. It is plausible that one population exhibits a greater affinity for the AWI, monopolizing its free space and shielding the other from interaction. However, this scenario does not seem to be applicable to CRP, as both the pentamer and decamer populations adopt preferred orientations in the absence of additives, indicating their interaction with the AWI. Furthermore, interactions and potential denaturation of a specific population at the AWI can alter the effective concentration of the solution, potentially causing a shift in the pentamer–decamer equilibrium. These factors are important in determining the behaviour of the protein on the grids, but in this study we did not consider these effects and analysed both the pentamer and decamer populations independently. The negatively charged B-face of CRP in the absence of additive (as shown in Supplementary Fig. S7*b*) is less sampled in the case of the CRP pentamer and is not sampled at all in the CRP decamer. When the surface charge at the AWI was changed to positive by addition of the cationic surfactant CTAB, the orientations changed in almost all cases, with a slight preference for certain alternate views (Fig. 3 ▸). Hence, electrostatic interactions at the AWI are an important factor in determining protein orientations in the absence of any additive. When non-ionic surfactants Tween 20 and Tween 80 are added to the sample buffer, the observed orientation distributions are uniform (Fig. 3 ▸). The orientation plots are similar for the non-ionic surfactants, as they all make the AWI charge neutral by occupying the free surface and act by diminishing the charge-based interactions between the protein and the AWI.

Although the addition of CTAB resulted in isotropic maps, the coverage of the 3D Fourier space was the least when compared with other surfactants (Table 3 ▸). The CTAB data sets showed a preference for one or more views, but in the macromolecules tested these preferences were desirable. Upon the addition of SLS, an even orientation distribution was observed for CRP pentamer, CRP decamer and catalase. Tween 20 and Tween 80 had similar effects on orientation, except in the case of CRP pentamer. A8-35 had an intermediate orientation distribution, which led to isotropic maps (Fig. 3 ▸). In the order of homogeneity of the orientation distributions, the surfactants can be ranked as SLS > Tween 20 = Tween 80 > A8-35 = CTAB. The map-versus-model FSCs were calculated for the data sets to obtain an independent resolution estimate, and they closely match the resolution estimated by comparison of the half-maps for most data sets (Supplementary Fig. S8 and Supplementary Table S2). Further, the use of different surfactants or sample preparation at different temperatures does not affect the structure of catalase, as shown by the density of heme in catalase (Supplementary Fig. S9).

Polyhistidine tag-dependent changes in orientation have recently been reported by Bromberg *et al.* (2022 ▸), but the mechanism by which this occurs remains unclear. The addition of a polyhistidine tag is a convenient method for expressing and isolating proteins (Hochuli *et al.*, 1988 ▸; Merchant *et al.*, 1998 ▸). Unlike in crystallography, it is intuitive to think that the flexibility of histidine tags does not pose a problem in structure determination by cryo-EM as the molecules are imaged individually, and the tag is averaged out during the reconstruction process if it is present in random conformations. At pH 8, which is more often used as the sample buffer, histidine should be deprotonated and exist as a hydrophilic polar group, and why this should cause a strong preference in orientation is not clear to us. The microenvironment at the AWI is complex and could be interacting with the histidine tag favourably and stabilizing it in that orientation.

Among the samples studied here, spike, PaaZ and β-galactosidase had a polyhistidine tag at the termini of the monomers. While the presence of the tag had no detrimental effect on the orientation distribution and reconstruction of PaaZ, it affected the orientations of the spike and β-galactosidase proteins significantly, and the addition of surfactants was not sufficient to mitigate the bias in the case of spike. In the case of PaaZ, the presence of a polyhistidine tag also causes a preference for the view that contains the affinity tag, but this is the side view of the protein, which contributes maximally to the reconstruction, and hence with very little sampling of alternate views the reconstruction is not as anisotropic as in spike. We note that the polyhistidine tag might cause the clumping observed for PaaZ and this remains to be tested. Further, the use of *D*3 symmetry in PaaZ and *D*2 symmetry in β-galactosidase compared with *C*1 symmetry in spike contributes to the quality of the final reconstruction. As the spike protein and β-galactosidase have been extensively studied and many structures have been reported, we wondered why it might be that we see such severe orientation bias and others do not. From the published structural studies on SARS-CoV-2 spike, it was realized that there have been variations in the reported specimens used for cryo-EM in terms of the expression system, the detergents added to the sample, the cleavage of tags, buffers *etc.* (Bangaru *et al.*, 2020 ▸; Bodakuntla *et al.*, 2023 ▸; Cheng *et al.*, 2023 ▸; Hardenbrook & Zhang, 2022 ▸; Wrapp *et al.*, 2020 ▸; Wrobel, 2023 ▸). The variability in the behaviour of the spike protein has been outlined in the review by Chua *et al.* (2022 ▸). They provide instances of the spike protein showing a preference for very thick ice (100 nm), displaying unfavourable behaviour on gold grids and exhibiting notably low particle concentrations on grids in the absence of a detergent (Chua *et al.*, 2022 ▸). Other parameters that might affect the orientation distribution include the glow-discharge settings for grids that change the hydrophilicity of the grid surface, the grid type (for example Quantifoil with carbon support or Ultrafoil with gold support), the grid hole diameter (0.6/1, 1.2/1.3 or 2/2), the freezing temperature and the blotting duration. In our case, removal of the affinity tag and addition of CTAB to the sample buffer were required to sample other views of the spike protein, which resulted in a map of reasonable resolution and improved isotropy (Fig. 5 ▸*b*). In the case of β-galactosidase, removal of the affinity tag was sufficient to obtain a high-resolution isotropic map without the need for any additive (Fig. 5 ▸*b*). We note that β-galactosidase with a C-terminal tag also showed preferred orientation in ice, as recently reported by Esfahani *et al.* (2024 ▸). Thus, the effect of the polyhistidine tag is a key factor to be tested when faced with orientation bias.

Furthermore, physical factors, such as the temperature of grid freezing and the grid type, can affect the mechanics of thin-film formation and evaporation/blotting, and in turn influence protein behaviour. We show that the temperature of grid freezing can change the orientation distributions of proteins by affecting the ice thickness and protein–protein interactions (Fig. 5 ▸). Additionally, varying only the hole diameter in a holey grid and keeping other factors constant led to a change in the preference of views in the case of catalase (Supplementary Fig. S2). In Quantifoil 1.2/1.3 grids, the approximate ratio of the area occupied by the hole to carbon is 38:62%, whereas in 0.6/1 grids the ratio decreases to 30:70%. This change can affect the protein distribution in the holes, carbon and AWI. It is now evident that this also significantly affects the orientation distribution of macromolecules in the thin films formed in the holes. We note the following two caveats in this study: (i) all of the grids used were from Quantifoil with amorphous carbon as the support film, and it is unclear whether similar behaviour will be observed with C-flat grids from Protochips that are manufactured differently or with other grids such as UltraFoil or NiTi alloy (Fan *et al.*, 2021 ▸; Quispe *et al.*, 2007 ▸; Russo & Passmore, 2016 ▸; Schürmann *et al.*, 2017 ▸), and (ii) only one sample (catalase) was tested and further extensive study with more samples is required to generalize the effect of the holey grid geometry.

Previous studies have used surfactants to determine the structures of the proteins examined in this study. A comparison of these results with our observations is presented below. Catalase has been used as a standard test sample in the context of the preferred orientation problem in two independent studies, which took different approaches to solve the problem. In an earlier study, several high-CMC detergents were screened, among which CHAPSO resulted in the best orientation distribution and map quality, although a high protein concentration of 30–40 mg ml^−1^ was used to obtain a good distribution of particles, and a small data set was sufficient to obtain a 2.2 Å resolution reconstruction by averaging 119 000 particles with an *E*_od_ of 0.76 (Chen *et al.*, 2022 ▸). Furthermore, the effects of ionic strength and pH were tested and were observed to have little or no effect on the protein orientation (Chen *et al.*, 2022 ▸). Our results, in an extension to this study, provide an understanding of the effects of surfactant head-group charge, temperature of grid freezing and hole carbon geometry on the orientations of catalase (Figs. 3 ▸ and 5 ▸ and Supplementary Fig. S2). In another study, nickel/titanium grids coated with 2D crystals of the hydrophobic protein HFBI (which shields the protein from exposure to the AWI) and 2.3 mg ml^−1^ catalase were used to prepare specimens and for subsequent data collection and structure determination (Fan *et al.*, 2021 ▸). The resulting catalase map has a resolution of 2.3 Å from 169 897 particles with an *E*_od_ of 0.80, which is comparable to our data sets (Table 3 ▸).

Similarly, multiple structures of CRP pentamer and decamer were reported in a study by Noone *et al.* (2021 ▸), which focused on the effects of pH and ligand addition on the complement-binding properties of CRP. One of the grid-freezing conditions reported was CRP apoprotein at pH 7.5 with 0.05% Tween 20 added and the grid held at 4°C and 65% humidity. These freezing conditions are similar to those described here, except for the concentration of Tween 20 used and the temperature and humidity during freezing. The orientations observed in this case look similar and the resulting reconstructions from 256 289 and 204 354 particles for the pentamer and decamer (*C*5 and *C*1 symmetry) resulted in resolutions of 3.2 and 2.8 Å, respectively. In comparison, we have obtained a 3.3 Å resolution map for the pentamer and a 4 Å resolution map for the decamer by averaging 25 000 and 51 000 particles, respectively (Table 3 ▸). In the field of cryo-EM, the greatest variation is observed in sample preparation, sometimes using the same instrument. Therefore, it is reassuring to observe that other independent investigations (along with different grid-freezing procedures) of catalase and CRP yield 3D reconstructions of comparable quality.

The AWI is a complex environment; the properties of the bulk and the surface vary drastically and it is an active area of research in the fields of surface chemistry and aerosols (Martins-Costa & Ruiz-López, 2023 ▸; Nguyen *et al.*, 2020 ▸; Zhong *et al.*, 2019 ▸). Only effects that occur at the surface can be observed, and trends can be analysed to improve sample-preparation methods. The explanation as to why they occur cannot be understood using standard cryo-EM experiments alone and requires complementary approaches such as infrared spectroscopy or dynamic surface-tension measurement studies (Carter-Fenk *et al.*, 2021 ▸; Tang *et al.*, 2020 ▸). Alternative approaches to sample preparation have been proposed in recent years to overcome the preferred orientation issue (Esfahani *et al.*, 2024 ▸; Drulyte *et al.*, 2018 ▸; Glaeser & Han, 2017 ▸; Jain *et al.*, 2012 ▸). One such approach was recently reported by Huber and coworkers, in which the sample was filled with nanosized capillaries made of silicon-rich nitride membranes embedded on a chip that physically controls the ice thickness. No blotting is involved in this process and some test samples have demonstrated the potential of this method (Huber *et al.*, 2022 ▸). However, the AWI is replaced by the solid–water interface, which may contribute some effects of its own. The further use and standardization of such methods are required to understand their general applicability.

## 5. Conclusion and outlook

One of the goals of single-particle cryo-EM is to collect a smaller data set and average a minimum number of particles to obtain a high-resolution reconstruction (Henderson, 1995 ▸). However, in reality many factors affect this, including the sample heterogeneity, detectors, beam-induced motion *etc.,* and the recently observed effects of the AWI and preferred orientation can be added to this list. We performed a comprehensive examination of how macromolecule orientations respond to alterations in physical factors, such as freezing temperature, and chemical factors, such as the addition of surfactants or the presence of affinity tags, during grid freezing for a standard set of proteins. This analysis provides insights into the behaviour of proteins on grids and can be utilized to address the preferred orientation problem systemically for any given macromolecule. When using surfactants, it is crucial to carefully assess protein stability using other techniques such as native gel electrophoresis, differential scanning fluorimetry or size-exclusion chromatography before data collection to save time and resources. Furthermore, our findings highlight the necessity to innovate and create small-molecule additives that are inert to biological samples and can effectively occupy the AWI and alleviate its deleterious effects on proteins.

## Supplementary Material

PDB reference: CRP pentamer with CTAB, 8wv4

PDB reference: CRP decamer with CTAB, 8wv5

PDB reference: PaaZ with CTAB at 4°C, 8wv6

PDB reference: catalase with SLS, 8wzh

PDB reference: spike with CTAB, 8wzi

PDB reference: catalase at 20°C, 8wzj

PDB reference: catalase at 4°C, 8wzk

PDB reference: catalase with CTAB, 8wzm

EMDB reference: CRP pentamer with CTAB, EMD-37864

EMDB reference: CRP decamer with CTAB, EMD-37865

EMDB reference: PaaZ with CTAB at 4°C, EMD-37866

EMDB reference: catalase with SLS, EMD-37952

EMDB reference: spike with CTAB, EMD-37953

EMDB reference: catalase at 20°C, EMD-37954

EMDB reference: catalase at 4°C, EMD-37955

EMDB reference: catalase with CTAB, EMD-37956

EMDB reference: β-galactosidase, no tag, EMD-39808

EMDB reference: β-galactosidase, with tag, EMD-39809

Supplementary Figures and Tables. DOI: 10.1107/S2059798324005229/rr5238sup1.pdf

## Figures and Tables

**Table 1:** Summary of the parameters for data sets collected under different conditions All imaging was performed in counting mode.

Protein	Condition	Grid type (Quantifoil)	Buffer composition	Protein concentration (mg ml^−1^)	Detector	Box size (pixels)	Pixel size
CRP pentamer and decamer	No additive	1.2/1.3	20 m*M* Tris pH 8, 280 m*M* NaCl, 5 m*M* CaCl_2_, 0.03% NaN_3_	2.1	Falcon 3	256	1.07
	CTAB	1.2/1.3		2.6	Falcon 3	256	1.07
	SLS	1.2/1.3		2.6	K2	320	1.08
	Tween 20	1.2/1.3		3.6	K2	320	1.08
	Tween 80	1.2/1.3		6.8	K2	320	1.08
	A8-35	1.2/1.3		3.6	Falcon 3	320	1.07
Catalase	No additive, 20°C	1.2/1.3	50 m*M* Tris pH 8	0.625	Falcon 3	256	1.07
	CTAB	1.2/1.3		3.4	K2	320	1.08
	SLS	1.2/1.3		3.4	Falcon 3	256	1.07
	Tween 20	1.2/1.3		3.4	K2	320	1.08
	Tween 80	1.2/1.3		4.1	Falcon 3	320	1.07
	A8-35	1.2/1.3		3.4	Falcon 3	320	1.07
	4°C	1.2/1.3		0.625	Falcon 3	256	1.07
	37°C	1.2/1.3		0.625	Falcon 3	320	1.38
	4°C	0.6/1		0.625	Falcon 3	320	1.07
	20°C	0.6/1		0.625	Falcon 3	256	1.07
PaaZ	No additive, 4°C	0.6/1	25 m*M* HEPES pH 7.4, 50 m*M* NaCl	0.8	Falcon 3	320	1.07
	No additive, 20°C	0.6/1		0.8	Falcon 3	256	1.07
	No additive, 37°C†	0.6/1		0.8	Falcon 3	—	1.38
	CTAB, 4°C	0.6/1		0.8	Falcon 3	256	1.07
Spike	With tag, no additive	0.6/1	50 m*M* Tris pH 8, 200 m*M* NaCl, 1 m*M* DTT	1	Falcon 3	256	1.07
	With tag, with CTAB	0.6/1		1.3	Falcon 3	320	1.07
	Without tag, no additive	0.6/1		2	Falcon 3	256	1.07
	Without tag, with CTAB	0.6/1		2	Falcon 3	256	1.07
β-Galactosidase	With tag, no additive	0.6/1	100 m*M* Tris pH 8, 200 m*M* NaCl, 5 m*M* CaCl_2,_ 2.5 m*M* MgCl_2_, 2 m*M* β-ME	5	Falcon 3	320	1.07
	Without tag, no additive	0.6/1		5	Falcon 3	320	1.07

† No data were collected due to higher order structures of particles.

**Table 2:** Properties of the surfactants used in this study

Additive	Charge	Ionic or non-ionic	CMC†	Concentration used	Aggregation number	Molecular weight (Da)†	Alkyl-chain length	Saturation in alkyl chain
CTAB	Positive	Ionic	1 m*M* (0.04%)	0.054 m*M* (0.002%)	170	364	16	Saturated
SLS	Negative	Ionic	14.6 m*M* (0.42%)	1.37 m*M* (0.04%)	—	293	12	Saturated
Tween 20	Neutral	Non-ionic	0.06 m*M* (0.007%)	0.04 m*M* (0.005%)	80	1228	12	Saturated
Tween 80	Neutral	Non-ionic	0.012 m*M* (0.002%)	0.038 m*M* (0.005%)	58	1310	18	Unsaturated
A8-35	Negative	Ionic	NA	0.01%	NA	∼9000	NA	NA

† CMC and molecular weight values are from the Anatrace and Sigma Aldrich webpages.

**Table 3:** Comparison of parameters for no-additive and surfactant-additive data sets

Protein	Condition	No. of particles	Resolution (Å) (half-map FSC 0.143)	Efficiency of Fourier space coverage	Sphericity
CRP pentamer	No additive	14601	18	0.78	NA
	CTAB	36353	3.3	0.80	0.98
	SLS	31699	4.2	0.80	0.97
	Tween 20	25674	3.3	0.80	0.86
	Tween 80	32330	7.5	0.69	NA
	A8-35	26737	10	0.78	NA
CRP decamer	No additive	9419	20	0.52	NA
	CTAB	25992	3.5	0.85	0.98
	SLS	59211	3.7	0.79	0.97
	Tween 20	51784	4.0	0.78	0.92
	Tween 80	36870	4.2	0.79	0.76
	A8-35	104369	3.5	0.78	0.98
Catalase	No additive	138000	2.7	0.72	0.96
	CTAB	153336	2.8	0.76	0.97
	SLS	33241	3.7	0.80	0.98
	Tween 20	88395	2.9	0.78	0.98
	Tween 80	92163	2.9	0.80	0.96
	A8-35	122000	3.1	0.77	0.98
PaaZ	No additive	51393	4.0	0.76	0.80
	CTAB	89454	2.3	0.75	0.98
